# Linkage of primary care prescribing records and pharmacy dispensing Records in the Salford Lung Study: application in asthma

**DOI:** 10.1186/s12874-020-01184-8

**Published:** 2020-12-10

**Authors:** Holly Tibble, James Lay-Flurrie, Aziz Sheikh, Rob Horne, Mehrdad A. Mizani, Athanasios Tsanas, The Salford Lung Study Team

**Affiliations:** 1 Usher Institute, University of Edinburgh, Bioquarter 9, 9 Little France Road, Edinburgh, Scotland EH16 4UX; 2 Asthma UK Centre for Applied Research, Bioquarter 9, 9 Little France Road, Edinburgh, Scotland EH16 4UX; 3 GlaxoSmithKline UK Ltd, Brentford, UK; 4 Health Data Research U004B, Edinburgh, UK; 5 Centre for Behavioural Medicine, UCL School of Pharmacy, London, UK

## Abstract

**Background:**

Records of medication prescriptions can be used in conjunction with pharmacy dispensing records to investigate the incidence of *adherence*, which is defined as observing the treatment plans agreed between a patient and their clinician. Using prescribing records alone fails to identify primary non-adherence; medications not being collected from the dispensary. Using dispensing records alone means that cases of conditions that resolve and/or treatments that are discontinued will be unaccounted for. While using a linked prescribing and dispensing dataset to measure medication non-adherence is optimal, this linkage is not routinely conducted. Furthermore, without a unique common event identifier, linkage between these two datasets is not straightforward.

**Methods:**

We undertook a secondary analysis of the Salford Lung Study dataset. A novel probabilistic record linkage methodology was developed matching asthma medication pharmacy dispensing records and primary care prescribing records, using semantic (meaning) and syntactic (structure) harmonization, domain knowledge integration, and natural language feature extraction. Cox survival analysis was conducted to assess factors associated with the time to medication dispensing after the prescription was written. Finally, we used a simplified record linkage algorithm in which only identical records were matched, for a naïve benchmarking to compare against the results of our proposed methodology.

**Results:**

We matched 83% of pharmacy dispensing records to primary care prescribing records. Missing data were prevalent in the dispensing records which were not matched – approximately 60% for both medication strength and quantity. A naïve benchmarking approach, requiring perfect matching, identified one-quarter as many matching prescribing records as our methodology. Factors associated with delay (or failure) to collect the prescribed medication from a pharmacy included season, quantity of medication prescribed, previous dispensing history and class of medication. Our findings indicate that over 30% of prescriptions issued were not collected from a dispensary (primary non-adherence).

**Conclusions:**

We have developed a probabilistic record linkage methodology matching a large percentage of pharmacy dispensing records with primary care prescribing records for asthma medications. This will allow researchers to link datasets in order to extract information about asthma medication non-adherence.

## Background

Medication data can be used in research to assess changes in medication prescribing trends over time [1], for pharmacovigilance studies, and to investigate patients not adhering to the treatment plans agreed upon with their General Practitioner (GP) [2–4]. Investigating medication data enables researchers to estimate the frequency, burden, and costs of non-adherence [5–7], identify the most at-risk to suboptimal clinical outcomes, evaluate the effectiveness of adherence interventions [8–10], and appropriately adjust for the impact of non-adherence on safety and efficacy data in clinical trials [11, 12].

In studies of linked (or integrated) prescribing and dispensing records, failure to collect the initial asthma prescription (*primary non-adherence*) has reported incidence between 12 and 45% [13–17], with high variance due to differences in the right censoring point. Studies across multiple chronic conditions reported a pooled general primary non-adherence rate of 9–17% [18–20].

In England, prescribing and dispensing of medications are recorded by separate processes. After a medication prescription is issued to a patient by a GP or another authorized prescriber [21], the prescription is taken to a dispensing outlet such as a community pharmacy [22]. When the prepared medicine is released to the patient, details relating to payment for medications are recorded and managed by the NHS Business Services Authority (NHSBSA). While analysis of medication adherence can be estimated using either the GP’s prescribing records or the NHSBSA medication dispensing records alone, there are limitations to each approach. Without linking the records together, it is not possible to ascertain whether a prescribed medication was collected, or to rule out other reasons for irregularities in collection such as treatment conclusion or sanctioned treatment interruptions [1, 23, 24].

Since 2015, NHSBSA dispensing data have included a patient identifier (NHS number) [25]; this is, however, not routinely linked to primary care prescribing records held by Public Health England (PHE). The NHSBSA and PHE records also do not have a common unique prescribing event identifier. Therefore, even with a data sharing agreement in place, matching records (one-to-one) using common identifiers (known as *deterministic linkage*) is currently impossible.

Therefore, it is necessary to link records probabilistically; estimating the likelihood that two records will match given the data they contain. Neither pharmacy nor primary care records are written with future linkage in mind, and as such they often require substantial pre-processing. The quality of the data linkage can be improved by integrating domain knowledge to identify non-matching but equivalent values, for example converting between units of dose strength.

The distinction between what should be considered deterministic or probabilistic is often disputed, as even complex probabilistic linkage processes can be broken down into their rule-based components and both linkage types can allow for imperfect (or *fuzzy*) matching on certain features [26], such as the dates of events in our case (which we would not expect to match all the time). The nature of administrative data source linkage, such as with Electronic Health Records, necessitates the use of fuzzy matching to overcome such prevalent qualities as missing data, free-text values, non-standardised units, and generic medication substitutions (resulting in different medication names). There are cases in which deterministic linkage will not only reduce the overall accuracy of the linkage, but may also introduce bias [27, 28].

Padmanabhan et al. have previously demonstrated the methodology used for linking UK health datasets when the unique patient identifier (NHS number) contained missing and erroneous values prohibiting deterministic linkage, including the creation of a ranking system for candidate links based on the matching information between them [29].

## Methods

### Aim

The linkage of prescribing and dispensing records can enable the extraction of information about adherence to prescribed medications, including the identification of uncollected medications. In this study, we sought to develop a novel methodology linking primary care prescribing and dispensing records without a common identifier, using heuristics and features extracted from free-text fields.

The GUILD [30] and RECORD [31] guidelines for data linkage reporting were applied where necessary information was not reported elsewhere [32–34]).

### Data source

The Salford Lung Study (SLS) was a prospective, 12-month, open-label, parallel group, randomised controlled trial (RCT) conducted in 74 general practice clinics in Salford and South Manchester, UK [35]. A total of 4233 participants with asthma were recruited in primary care settings by the healthcare professionals who provided their normal everyday care, and randomly allocated to either initiate a combination fluticasone furoate/vilanterol treatment or to continue their maintenance therapy (“usual care”).

Participants were at least 18 years old at the time of recruitment, with a clinical diagnosis of symptomatic asthma made by a GP and had to be taking regular maintenance inhaler therapy with Inhaled CorticoSteroids (ICS) either alone or in combination with a Long-Acting β_2_-Agonist (LABA). The main exclusion criteria were a recent history of life-threatening asthma, a history of Chronic Obstructive Pulmonary Disease (COPD), or concomitant life-threatening disease [34, 36]. Many of the participants in the study cohort would have been excluded from conventional RCTs due to their multi-morbidities [33, 36], which increased the representativeness of the study cohort to the target population.

The trial was registered in the National Institute of Health’s database of clinical studies [32] (clinicaltrials.gov identifier NCT01706198). The study was conducted in accordance with the standards dictated by the National Research Ethics Service Committee North West (reference 12/NW/0455), as well as the International Conference on Harmonisation, Good Clinical Practice, all applicable data protection requirements and the ethical principles outlined in the Declaration of Helsinki 2013.

### Data format

The dispensing data contained 225,235 records, for 4197 unique participants, between 27th November 2012 and 9th December 2016. The prescribing dataset contained 339,792 records for 4233 unique participants between 22nd November 2012 and 17th January 2017, however records outside of the dispensing data period were excluded.

Both datasets contained a (common) subject ID, free text drug description, date (prescription or dispensing, respectively), the dose strength, dose instructions, and a numeric quantity of medication prescribed (e.g. “200 dose inhaler”). Between the two datasets, there were 8291 unique (*free text*) drug descriptions.

### Inclusion and exclusion criteria

All unique drug descriptions, in either the prescribing or dispensing records, were searched for the presence of one or more of the keywords listed in Appendix A. From here, the drug classes were assigned: Short-Acting β_2_-Agonist (SABA), Long-Acting Muscarinic receptor Antagonist (LAMA), LABA, theophylline, ICS, LeukoTriene Receptor Antagonist (LTRA), cromoglicate, steroid, or immuno-suppressant. If only one candidate class was identified, the drug class was coded according to the drug class keyword. A drug was coded as an ICS and LABA combination medication (ICS + LABA) if active ingredients of both ICS and LABA varieties were flagged, a SABA if a medicine containing both SABA and LAMA ingredients were flagged. Medications that did not match any of the keywords in Appendix A were considered to be non-asthma medications and were removed. A medication class keyword was generated, containing a composite of the active ingredients, to be used in the matching algorithm.

Furthermore, drug descriptions were searched for any of the exclusion keywords and brand names listed in Appendix B, which signalled that a medication was being used for an indication other than asthma (such as nasal spray corticosteroids for rhinitis).

### Variable recoding

Several free text variables were recoded using custom look-up tables, to allow semantically identical, but syntactically variant (such as “128mcg” vs “128 micrograms”, and other type abbreviations and variations) records to be aligned. Of note, we modified the recorded medication quantity to estimate the number of doses (puffs), rather than the number of units (inhalers). This variable integrates domain knowledge of the number of doses per unit for each medication strength combination (high potency medications are often dispensed at lower volumes), calculated using the most common volumes in the data. In order to avoid candidate links being ruled out as potential matches on the basis of our quantity variable modifications, we included a so called ‘alias’ quantity [27], to be considered if the ‘primary’ quantity values did not match. The process is summarized in Appendix C.

### Identification of duplicates

Duplicates of prescribing and dispensing records are common due to errors in data entry [37–39]. Duplicate records in the data would have a strong adverse effect on the matching algorithm, as it would be forced to incorrectly match distinct records in one set to duplicates in the other. We identified duplicate records by searching for commonalities within the same person, date (dispensing or prescribing respectively), medication brand name, and medication (active ingredient) keyword, in addition to the following combinations of (modified) variables:

- Matched on quantity and dose
- Matched on dose, and the quantity was not matched due to data missingness
- Matched on quantity, and the dose was not matched due to data missingness.

### Data linkage

The datasets of prescribing and dispensing records were merged such that a record (a *candidate link*) was generated for each eligible (common patient identifier and medication class) pair of records for matching. We note that the medication class keyword, composed of the active ingredients identified, was used in the place of a brand name such that generic substitutions would be identified as appropriate candidates for matching records. Pairs of records were eligible if the suggested dispensing date occurred after the prescription was written, but no more than 6 months *after* the prescription was written, at which point the prescription became invalid.

Probabilistic linkage, which aims to match records based on multiple non-unique features, utilizes *weights* to determine the strength of a link. These weights are numerical values representing the similarity of two records, derived using domain knowledge about the prevalence of dissimilarities between features in true matches.

In this linkage, a rule-based approach, based on a simplified posterior multivariate distribution of clerically reviewed data and previous literature, was used to weight candidate links for estimated likelihood of being a true match. Candidate links could then be ranked, and those with a linkage weight (calculation detailed in Appendix D) less than 70% excluded (combinations of features by match status that resulted in inclusion are listed, along with their sum weights, in Appendix E).

Generic substitution for brand named medications are common (when permitted by the prescriber, known as *open generic prescribing*) in asthma controller medications [15, 40, 41]. As such, brand name was assigned a lower maximum feature weight (20%) than the dose strength (35%, which will vary only when one record has a missing value, or in the rare case that a generic substitution requires a slightly different dosage) and quantity (35%, varying when a quantity was both uncommon and missing, and was imputed with a more prevalent but incorrect value). The final 10% weight corresponded to the time between the prescribing and dispensing events. Prescriptions issued less than 1 month prior to the dispensing were awarded the additional 10% weight, in line with the findings by Williams et al. that 95% of asthma prescriptions are filled within this time window [14], however a higher weight was not implemented due to the use of the time between weights in the final match selection process. That is, each set of dispensing records for each person-medication combination were looped through from the last to first through, as follows:

1. Identified the candidate in which the dispensing record occurs most recently after the prescription was written (record with highest match weight chosen if two candidate links on the same day were identified); this is a match between records,
2. Removed all other candidate links which contain the dispensing record or the prescribing records relating to this match,
3. Progressed to the previous dispensing for this person-medication.

**Fig. 1:**
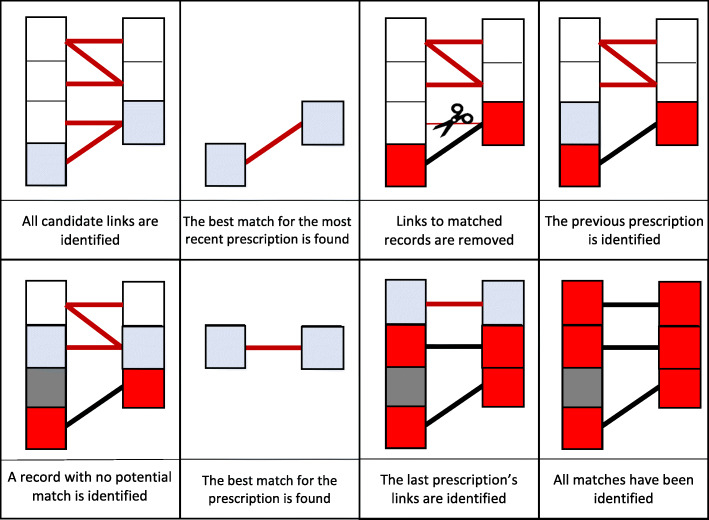
Diagram representing the data linkage algorithm

This process, illustrated in Fig. 1, is also described in more detail in Appendix F.

The most recent prescribing record before the dispensing was prioritised over more distant records with a higher match weight, as we considered it more likely that prescription records for the same person within such a short time window were for the same medication, recorded differently, rather than a new treatment.

Prescriptions that did not match any dispensing record were marked as unclaimed. We also noted dispensing records that were not matched (implying no corresponding prescription event) to assess linkage quality.

### Statistical analysis plan

As per the recommendations by Harron et al., the characteristics of the matched and unmatched records were compared in order to identify potential sources of bias [42]. Specifically, the missingness for each variable used in the matching was compared between matched and non-matched records, factors associated with prescription collection were assessed (statistical methodology described below), and the sensitivity of the algorithm parameters was tested by altering certain thresholds and requirements and comparing the proportion of records that were matched.

As well as estimating the incidence of primary non-adherence, we used our linked dataset to analyse factors effecting the collection of prescribed medications. By comparing our results to others using integrated health records (those that are linked, or linkable, inherently) we are able to demonstrate the validity of our linked dataset to answer epidemiological questions about high-risk individuals.

We used multivariate Cox survival analysis to assess the statistical relationship between the season of the prescription, the drug class of the prescription, the number of previously unclaimed prescriptions, and the strength and quantity of the medication prescribed, on the time between the prescription being written and dispensed. Survival analysis calculates the rates (*hazard rates*) of medications being collected at any specific time since the prescription was written. Comparing the ratios (*hazard ratios*) between two levels of a factor (such as male and female) allowed us to assess the difference that this factor made when everything else (age, medication, etc.) remained constant. Although a prescription could be dispensed up to 6 months after it was written, it is uncommon that their collection will be delayed for more than 7 days [14, 15]. Furthermore, a delay of beyond 1 month would likely result in a gap in medication availability and thus be considered poor adherence. As such, we wanted to find a threshold at which prescriptions could be recorded as ‘hitherto uncollected’, known as being right censored. We set this threshold at the minimum number of weeks such that fewer than 2% of subsequently collected prescriptions would be right censored.

### Naïve benchmarking

We compared our results to those produced from a simplified algorithm in which records were pseudo-deterministically matched, such that candidate links required perfect matching on medication name, dose, quantity, and dose directions, without any variable recoding or removal of duplicate records. The date variable, however, still allowed flexible matching as medications can be dispensed up to 6 months following prescription.

The same iterative linkage procedure was used in the algorithm detailed previously, without the inclusion of the linkage weights as a tiebreaker between candidate links on the same day.

As the dose directions were long, free-text strings, written separately by both the prescribing and dispensing agents, we also repeated the benchmarking analysis, with imperfect matching on the dose directions permitted.

Links identified by this process should not be considered the ground truth, or the gold standard, as the algorithm will default to match records which are more distanced chronologically but similar syntactically, rather than semantically similar and chronologically closer record matches which are more likely to be estimated by the full algorithm. As such, the matches identified between approaches will not be directly compared.

### Reporting

This study has been reported in accordance with the GUILD and RECORD reporting guidelines [30, 31].

## Results

### Data cleaning

**Fig. 2:**
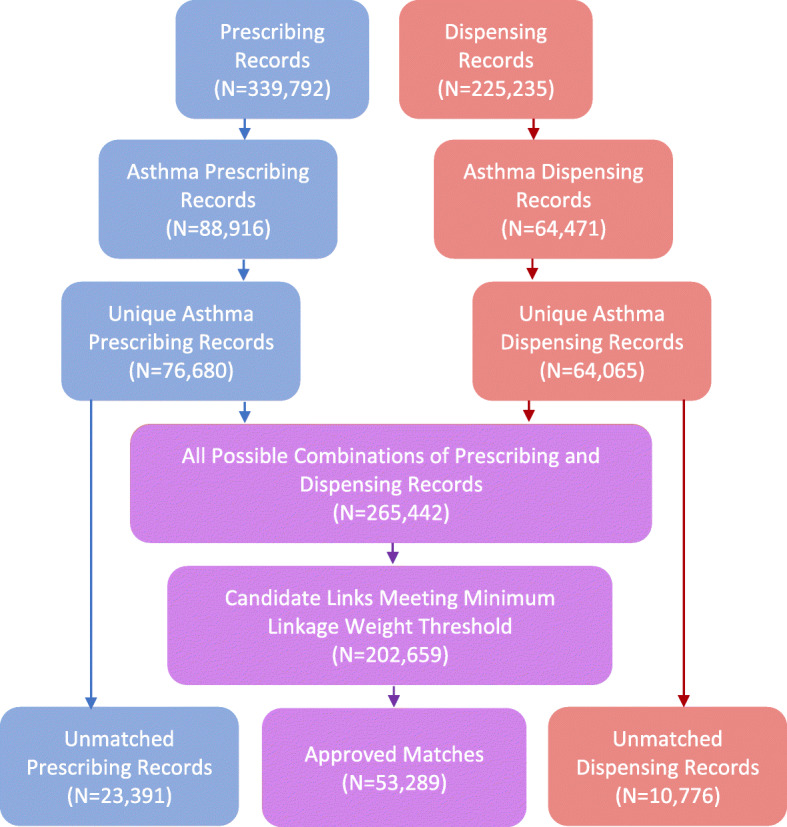
Data Linkage Flow Diagram

Of the 8291 unique drug descriptions, 928 (11%) were identified as relating to asthma medications (list of keywords used in string search provided in Appendix A). Searching the drug descriptions for the set of exclusion keywords led to the removal of 71 (8%) further records (list and frequency of keywords in Appendix B). Removing the excluded medications left 88,916 prescribing records and 64,471 dispensing records (Fig. 2). Finally, duplicates were removed (12,236 prescribing records and 406 dispensing records), leaving 76,680 prescribing records (86%) and 64,065 dispensing records (99%).

### Matching

The full join on the prescribing and dispensing records generated 265,442 candidate links for linkage weight assessment (Appendix D). Sixty two thousand and seven hundred eight-three candidate links were removed (23.7%) as they did not fulfil the minimum linkage weight threshold, leaving 202,659 candidates to be sorted through the matching algorithm. After the algorithm was applied, 53,289 candidate links were confirmed as matches: 69.5% of prescribing records (*n* = 76,680), and 83.2% of dispensing records (*n* = 64,065).

As shown in Fig. 1: Diagram representing the data linkage algorithm.

**Fig. 3:**
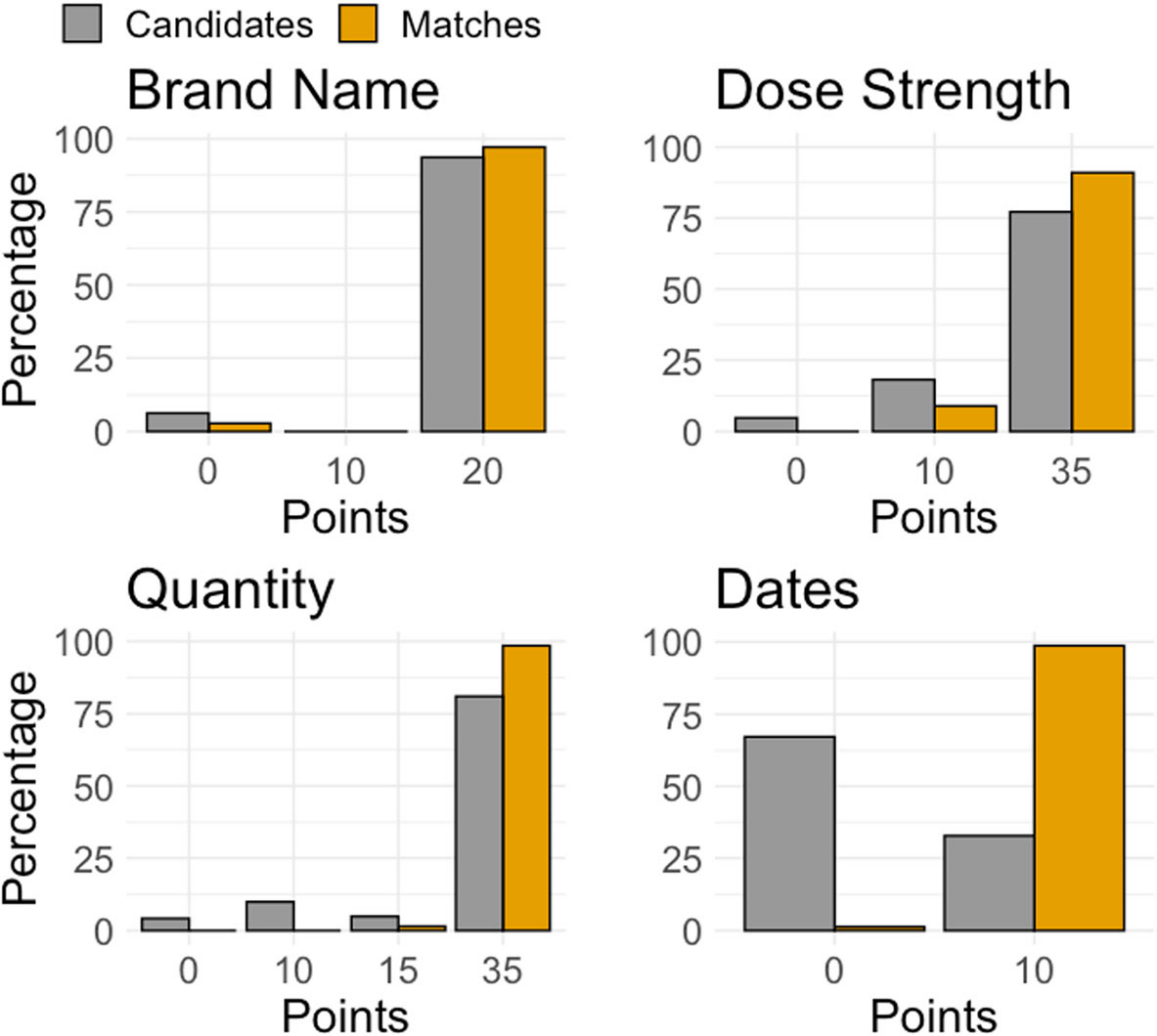
Distributions of linkage weight points per variable, for candidates and final matches

Figure 3, there was a substantial discrepancy between the time between the prescribing and dispensing for the candidate links and the matches, with 99% of matches having less than one month between prescribing and dispensing (compared to 33% of candidate links).

The median percentage of prescriptions claimed by an individual was 79%, with an interquartile range of 50–92% (range 0–100%). 23% of individuals claimed fewer than 50% of their prescriptions.

### Quality assurance

We inspected 23,391 prescribing records (31%) and 10,776 dispensing records (17%) for which a match could not be made (including those with candidate links which were not matched by the matching algorithm). In the non-matched prescriptions, 9% (*n* = 2109/23,391) had missing medication dosage, and < 1% (*n* = 87/23,391) had missing data on quantity (both missing in less than < 0.1%). In the non-matched *dispensing* records, however, it was 62% (*n* = 6639/10,776) and 58% (*n* = 6222/10,776), respectively (both missing in 55%).

### Survival analysis

**Fig. 4:**
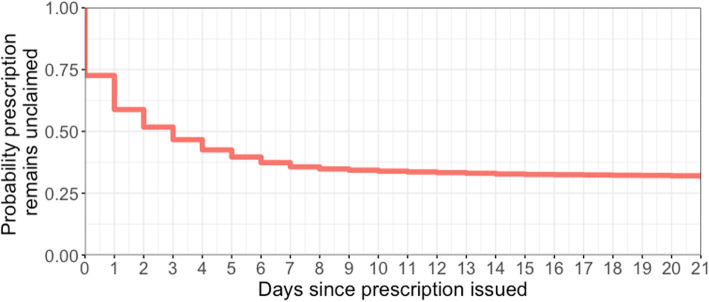
Kaplan-Meier of the time to collecting prescriptions, censored at three weeks

31% of prescriptions (*n* = 23,391) were labelled as unclaimed. In claimed prescriptions (*n* = 53,289), the median time between the prescription being written and the medication being dispensed was 1 day (upper-lower inter-quartiles = 0–3 days), and fewer than 5% of people took longer than 1 week to claim (0.9% longer than 30 days). Considering uncollected prescriptions to be right-censored at 6-months, at which point the prescription expires, the median time to collection was 3 days (upper-lower inter-quartiles = 0–178 days; Fig. 4).

The multivariate Cox survival analysis model included 76,584 prescription records – having removed 96 with missing quantity. The prescriptions were claimed in 52,186 of these records, with less than 2% being collected beyond 3 weeks after the prescription was issued. As such, 21 days was set as our right censoring point. We found a lower hazard of claiming medications in summer (June–August: 3% decrease, 95% CI = 1–6%) compared to spring (Table 1), indicating that they were claimed slower in summer than in spring. There was no statistically significant difference in the claiming of medications between spring and winter or spring and autumn. Higher quantities (by number of doses) of prescribed medications were associated with modest reduction in hazard of collecting the medication (*p* < 0.001). Finally, proportions of previous prescriptions that were unclaimed (categorized into tertiles) were a strong predictor – with medium vs low tertiles hazard ratio of 0.57, and high vs low of 0.20 (*p* < 0.001). Rescue medication (SABA and steroids) had the highest hazard rates (1.433 and 1.839, respectively). Of the controller medications, those associated with higher asthma severity (according to the British Thoracic Society (BTS) treatment steps [43]), such as LAMA and LTRA medicines, had higher hazards than lower severity treatments such as ICS and combination ICS + LABA medications.

**Table 1:** Cox Proportional hazards model risk factors associated with time to collecting a prescribed medication

	Hazard Ratio (95% Confidence Interval)	Statistical significance (***p***-value)
Season		
Spring	{reference}	
Summer	0.967 (0.944–0.991)	0.008 ^*^
Autumn	0.981 (0.958–1.005)	0.123
Winter	1.003 (0.979–1.028)	0.791
Drug Class		
SABA	1.433 (1.387–1.479)	< 0.001 ^*^
LABA	0.938 (0.890–0.990)	0.019 ^*^
ICS	{reference}	
ICS + LABA	1.067 (1.033–1.102)	< 0.001 ^*^
Cromoglicate	0.778 (0.389–1.558)	0.479
Immuno-suppressants	1.244 (1.100–1.408)	< 0.001 ^*^
LAMA	1.349 (1.161–1.567)	< 0.001 ^*^
LTRA	1.350 (1.289–1.414)	< 0.001 ^*^
Theophylline	1.040 (0.897–1.205)	0.604
Oral steroids	1.839 (1.743–1.940)	< 0.001 ^*^
Previously unclaimed medications		
Low tertile	{reference}	
Mid tertile	0.565 (0.553–0.577)	< 0.001 ^*^
High tertile	0.198 (0.193–0.204)	< 0.001 ^*^
Quantity of doses prescribed	1.000 ** (1.000–1.000)	< 0.001 ^*^

Statistically significant variables (using a threshold of *p* = 0.05) are denoted by a star (^*^)

^**^ Coefficient 0.9999 to four decimal places, and therefore lower than the reference value

### Naïve benchmarking

There were 88,916 prescribing records and 64,471 dispensing records identified relating to an asthma medication (without the removal of duplicates). Of these, 584 (0.7% of prescribing records and 0.9% of dispensing records) were pseudo-deterministically linked. Even when imperfect matching on dose-directions was permitted, only 15.4% of prescribing records and 21.2% of dispensing records could be matched (*n* = 13,698 matches).

## Discussion

We have developed a novel methodology matching prescribing and dispensing electronic health records and demonstrated this led to matching 70% of asthma prescribing and 83% of dispensing records. Fewer than 5% of prescriptions were eventually claimed after one week of the issuing of the prescription. 30% of prescriptions were labelled as uncollected.

The key strength of this study is the variety of integrated mechanisms – incorporating domain knowledge relating to asthma medications (such as semantic harmonization from brand name to active ingredients) and rule-based natural language feature extraction and harmonization (such as converting a free-text dose to a numeric value with common units).

Using a naïve benchmarking algorithm that required perfect matching between prescribing and dispensing records (except for the date variable; pseudo-deterministic linkage), we were able to demonstrate the superiority of our proposed methodology. In this benchmark linkage, only 15% of the prescribing records and 21% of dispensing records were matched, even when imperfect matching on free-text dose directions was permitted. This was a result of syntactically variant (different formats and value units) but semantically matching data between the two sources of information.

We identified a set of records for dispensed medications (17%) for which no matching prescribing record was identified. In the non-matched dispensing records, 62% had missing medication strength, and 58% had missing quantity. In its current state, the algorithm will not match records with high amounts of missing data even if no other match is identified.

In Appendix D, we see that 3% of matches had distinct and non-missing medication brand names. This highlights that potentially brand substitutions occurring at the pharmacy need to be accounted for in the matching [44]. The variable with the biggest change in distribution between the candidate links and the final matches was whether the medication was dispensed within one month of prescribing – 33% of candidates and 99% of matches (see Fig. 1: Diagram representing the data linkage algorithm.

Figure 3). In fact, we found that only 1% of prescriptions were claimed more than a month after the prescription was written.

Our finding that 30% of prescriptions were labelled as uncollected, known as primary non-adherence, was a substantially higher proportion than the 8–20% found in previous asthma studies in US administrative health data studies [13–15, 41, 45]. One might assume that subsidised prescriptions, as we have in England, would result in higher primary adherence rates, as a barrier to adherence has been removed. On the contrary, a recent study in Canada, where prescriptions are subsidised and thus considerably more affordable than in the USA, found that the fill rate for new asthma prescriptions was only 69% in adults [16]. As such, future work must be conducted in order to find cost-effective interventions to reduce primary non-adherence in asthma.

As there is no true linkage event identifier (person-prescription), it is not possible to compare our identified matches to some ground truth, a common limitation highlighted in the aforementioned linkage quality assessment guidelines by Harron et al. [42]. As the benchmarking analysis allowed prescribing and dispensing date variables to differ, hence pseudo-deterministic, even this does not identify ‘perfect matches’ between records. If the ground truth was known, it would be possible to compare directly the matches estimated from the benchmark and pseudo-deterministic analyses and evaluate how well our algorithm improves the matching quality. While the ground truth may not be possible to determine in challenging real-world data, even with manual review, one could also perturb data in which the ground truth is known to closer approximate the real use case, and evaluate the algorithm’s accuracy. This would be a very interesting direction that future research work could investigate further, and would provide further insights in terms of confidence in the accuracy of the data linkage process.

In lieu of this, we conducted quality assurance comparing features of the matched and unmatched records, as recommended by Harron et al.’s guidelines [42]. We observed that prescriptions (for which the status of being non-matched might imply either medication non-initiation, or not being correctly matched using the proposed algorithm) had missed medication strength in fewer than 10% of records, and missing quantity in fewer than 1%. In the non-matched dispensing records (which should occur only in rare emergency prescriptions and indicate shortcomings in matching prescription and dispensing records), 62% had missing strength and 58% had missing quantity. This indicates that one of the biggest barriers to successful record linkage was poor medication dispensing record quality.

The frequency of non-matched dispensing records was our best indicator as to the quality of our linkage, however we found that 95% of these records that were missing quantity (58%) were also missing dose-strength. As such, reducing the weight threshold from 70 to 50%, would have had a substantial effect on the pool of candidate links allowed to be used in the matching algorithm. With so much missing data, however, the veracity of these matches would be hard to ascertain.

The strong influence of data quality on the success of the linkage algorithm makes it difficult to benchmark our results against other record linkage algorithms or even treatment initiation studies in populations with linkage conducted routinely. Comparisons to algorithms derived in other medication indications, such as in acute conditions such as tuberculosis, or in other chronic illnesses such as mental health conditions, are even harder. Furthermore, not all countries have a unique patient identifier, resulting in the use of demographic data such as gender, year of birth, and postcode, to identify entries belonging to the same person [46]. Regardless, we find other studies have reported similar levels of inconsistency between features in matched records, such as brand name, dose strength, and time between prescribing and dispensing [44, 47]. We also observed the substantial increase in matches when variables were cleaned, and recoded, and our probabilistic methodology was used in the place of a simple pseudo-deterministic matching.

As with all probabilistic matching approaches, and particularly in cases such as these with considerable number of missing entries and un-structured fields, it is possible that matches even with high assigned weights are incorrect. Indeed, it is not likely that the matches established in the benchmarking analysis are of higher accuracy than those in the primary analysis, and they cannot be directly compared. In future work, this algorithm should be tested in simulated data where the underlying ground truth is known for further validation, in order to better determine the accuracy of the linkage. There is potential that the design of the study on which this secondary analysis was conducted (a pragmatic randomised controlled trial) may have influenced the linkage in some way. Validating the proposed linkage algorithm in further additional randomised clinical trials would be needed to establish the generalizability of our findings.

In addition to testing in other datasets, in which the true links are known and can be compared to the estimated matches, further development of this study would be to test the sensitivity of the model to certain parameters such as the weights for each component, the degree of influence from the dates, and the minimum weight threshold. We remark that these intrinsic parameters can be seen as degrees of freedom that enable data modellers to explore different levels of certainty for record matching. At a higher level, these can be thought of as the equivalent free parameters which need to be explored and optimised for a given dataset: for example, in Support Vector Machines (SVM) one needs to optimise the penalty hyper-parameter (and depending on configuration additional hyper-parameters too). Consideration must also be taken to determine the acceptable limits of the false negative and positive rates, and the relative importance of the two, in specific settings. For example, in adherence studies, one might conservatively prefer to underestimate adherence than to overestimate it, and thus prioritise lowering the false positive rate.

Additionally, accounting for how much medication supply an individual currently has, or when their most recent previous prescription was issued, would allow the date component of the algorithm to correspond more meaningfully to the patient’s history. As previously discussed, matching may also be improved by the addition of an extension allowing candidate pairs for which one record had high amounts of missing data and no match was identified to be re-considered.

## Conclusions

The optimal dataset for measurement of medication non-adherence includes both prescribing records and dispensing records, such that prescriptions that are not collected from the dispensing agent and resolved/discontinued treatment regimens are accounted for. These are however seldom available. We therefore developed a novel methodology that matched 83% of pharmacy dispensing records to primary care prescribing records. In the 17% of dispensing records for which a match could not be identified, missing information was prevalent; particularly regarding the strength of the medication, and the quantity dispensed. A naïve benchmarking, requiring perfect matching, identified prescribing records for only 21% of the dispensing records. The presented methodology towards probabilistic record linkage enables preliminary assessment of whether patients are collecting their prescribed asthma medications and can improve clinicians’ understanding of patient adherence. Further external validation of these promising findings on additional datasets is needed given the uncertainty around linkage quality.

## Data Availability

The datasets analysed during the current study are not publicly available but are available by application to, and approval from, the Salford Lung Study scientific committee. Code scripts, in the R language, for all components of the data cleaning, linkage, and subsequent analysis will be made available in the open source GitHub website (https://github.com/hollytibble/Salford-Lung-Study_Adherence-Linkage).

## Appendix 1

**Table 2:** String Search Keywords by Medication and Drug Class Keyword Categories

Drug Class Keyword	Medication Keyword	String Search Keywords
SABA	SALBUTAMOL	“SALBUTAMOL”, “ALBUTEROL”, **“VENTOLIN”, “AIROMIR”, “SALAMOL”, “AIRSALB”, “SALAPIN”, “VENTMAX”, “ASMASAL”, “ESI-BREATHE”, “SALBULIN”, “SALIPRANEB”, “IPRAMOL”, “COMBIVENT”**
SABA	BAMBUTEROL	“BAMBUTEROL”, **“BAMBEC”**
LABA	FORMOTEROL	“FORMOTEROL”, **“FORADIL”, “FOSTAIR”, “SYMBICORT”, “FLUTIFORM”, “SPIROMAX”, “OXIS”, “ATIMOS”**
LABA	SALMETEROL	“SALMETEROL”, **“NEOVENT”, “SEREVENT”, “SERETIDE”, “SIRDUPLA”, “AIRFLUSAL”**
LABA	TERBUTALINE	“TERBUTALINE”, **“BRICANYL”**
LABA	TIOTROPIUM	“TIOTROPIUM”, “SPIRIVA”
LABA	VILANTEROL	“VILANTEROL”, **“RELVAR”,** “VILENTEROL”
LAMA	GLYCOPYRRONIUM BROMIDE	**“SEEBRI”**
LAMA	IPRATROPIUM	“IPRATROPIUM”, **“ATROVENT”, “RESPONTIN”, “IPRAVENT”, “SALIPRANEB”, “IPRAMOL”, “COMBIVENT”**
THEOPHYLLINE	THEOPHYLLINE	“THEOPHYLLINE”, **“NEULIN”, “SLO-PHYLLIN”, “UNIPHYLLIN”**
THEOPHYLLINE	AMINOPHYLLINE	“AMINOPHYLLINE”, “PHYLLOCONTIN”
ICS	BECLOMETASONE	“BECLOMETASONE”, **“ASMABEC”, “BECODISKS”, “CLENIL”, “QVAR”, “FOSTAIR”**
ICS	CICLESONIDE	“CICLESONIDE”, “ALVESCO”
ICS	BUDESONIDE	“BUDESONIDE”, **“BUDELIN”, “PULMICORT”, “SYMBICORT”, “SPIROMAX”**
ICS	FLUTICASONE	“FLUTICASONE”, **“FLIXOTIDE”, “FLUTIFORM”, “SERETIDE”, “SIRDUPLA”, “AIRFLUSAL”, “RELVAR”**
ICS	MOMETASONE	“MOMETASONE”, **“TWISTHALER”, “ASMANEX”**
LTRA	MONTELUKAST	“MONTELUKAST”, **“SINGULAIR”**
LTRA	ZAFIRLUKAST	“ZAFIRLUKAST”, **“ACCOLATE”**
LTRA	ZILEUTON	“ZILEUTON”, **“ZYFLO”**
CROMOGLICATE	NEDOCROMIL	“NEDOCROMIL”, **“TILADE”**
CROMOGLICATE	CROMOGLICATE	“CROMOGLICATE”, “CROMOGLYCATE”, **“INTAL”**
STEROID	OMALIZUMAB	“OMALIZUMAB”, **“XOLAIR”**
STEROID	PREDNISOLONE	“PREDNISOLONE”
IMMUNO-SUPPRESSANT	METHOTREXATE	“METHOTREXATE”, **“MAXTREX”, “METOJECT”, “METHOFILL”, “NORDIMET”, “ZLATAL”**
IMMUNO-SUPPRESSANT	CICLOSPORIN	“CICLOSPORIN”, **“CAPIMUNE”, “CAPSORIN”, “DEXIMUNE”, “NEORAL”, “SANDIMMUN”**
IMMUNO-SUPPRESSANT	AZATHIOPRINE	“AZATHIOPRINE”, **“IMURAN”**

String search keywords may appear under multiple medication and drug class keyword categories, if they contain more than one active ingredient, such as combination ICS LABA medications.

Bold string search keywords indicate brand names.

## Appendix 2

**Table 3:** Exclusion Keywords and Frequency

Exclusion Keyword	Unique Drug Descriptions (*N* = 928)
NASAL	39
NOSE	1
NOSTRIL	0
NASULE	0
HAYFEVER	0
EYE	11
EAR	0
DROP	16
TONGUE	0
FOAM	2
ENEMA	1
RECTAL	0
GASTRO ^*^	1
MODIFIED ^*^	0
CREAM	4
APPLY	0
SKIN	0
ULCER	0
OINTMENT	6
PATCH	0
CAPSULE^**^	2
SACHET	0
SPRAY	33
AZELASTINE	4
NASONEX	0
FLIXONASE	0
ANORA ELLIPTA	0
SUMATRIPTAN	0
AVAMYS	0
RHINOCORT	0
NASOBEC	0
NASOFAN	0
**TOTAL EXCLUDED**	71 (7.7%)

^*^ Excluding medications of drug class “steroid” or “theophylline”

^**^ Excluding medications of drug class “steroid”, “theophylline”, “tiotropium” or “glycopyrronium bromide”

## Appendix 3

### Variable Recoding

*Quantity Recoding:*

Quantities with values of over 28 were assumed to be the number of doses, rather than the number of units/inhalers. The most common recorded number of dose quantity was imputed as the most commonly occurring number of doses per unit (as the most common number of units prescribed is one) for that medication class. If the quantity was recorded in doses, this was set as the primary dose quantity, with the second most commonly occurring dose quantity as the alias value. If the quantity was recorded in units, the number of units multiplied by the most commonly occurring dose quantity was imputed as the primary value, and the second most likely as the alias.

*Dose Strength Recoding:*

All dose strengths were converted into upper case, spaces were removed, and the following string substitutions were made:

- “MICROGRAMS” replaced with “MCG”,
- “MICROGRAM” replaced with “MCG”,
- “MICROG” replaced with “MCG”,
- “UNITS” replaced with “U”

Strings were then searched for the first pattern of “0.5”, “500”, “400”, “320”, “200”, “184”, “160”, “125”, “100”, “92”, “80”, “50”, “25”, “20”, “10”, “5”, “4”, “2”, or “1”, followed by any of “MG”, “MCG” or “/”. ICS + LABA medications often recorded as X/X dose, in which the larger number relates to the ICS and the lower to the LABA. Some records listed the ICS + LABA combination medicines as ICS/LABA dose, and some as LABA/ICS dose; as such, the possible patterns were searched in order of size, rather than position in string.

## Appendix 4

**Table 4:** Linkage Weight Calculator

Factor	Criteria	Points	Factor Range	% of candidates	% of matches
Brand Name ^*^	Both records had non-missing, and distinct, brand names	0	0–20	6.3%	2.8%
	One or both of the records had a missing brand name	10		0%	0%
	Both records had non-missing, and matching, brand names	20		93.7%	97.2%
(Modified) Dose Strength	Both records had non-missing, and distinct, dose strengths	0	0–35	4.8%	0%
	One or both of the records had a missing dose strength	10		18.1%	9.0%
	Both records had non-missing, and matching, dose strengths	35		77.2%	91.0%
(Modified) Medication Quantity	Both records had non-missing, and distinct, primary and alias dose quantities	0	0–35	4.2%	0%
	One or both of the records had a missing primary quantity value, indicating that no value was observed or could be imputed	10		9.8%	< 0.1%
	Both records had non-missing, and distinct, primary dose quantities, but the alias of one record matched to the primary of the other	15		4.9%	1.5%
	Both records had non-missing, and matching, primary dose quantities	35		81.1%	98.5%
Date difference	Dispensing occurred more than one month after prescription (but less than six months)	0	0–10	67.2%	1.3%
	Dispensing occurred within one month of prescription	10		32.8%	98.7%

* If a generic medication was used, the brand name was listed as ‘generic’

## Appendix 5

**Table 5:** Included Feature Weight Combinations

WEIGHT	BRAND NAME	DOSE STRENGTH	QUANTITY	DATES
100	Non-missing and matching	Non-missing and matching	Non-missing and matching	Less than one-month delay
90	One or more missing	Non-missing and matching	Non-missing and matching	Less than one-month delay
	Non-missing and matching			More than one-month delay
80	Non-missing and distinct	Non-missing and matching	Non-missing and matching	Less than one-month delay
	Non-missing and matching		Primary/alias match	
	One or more missing		Non-missing and matching	More than one-month delay
75	Non-missing and matching	One or more missing	Non-missing and matching	Less than one-month delay
		Non-missing and matching	One or more missing	
70	Non-missing and distinct	Non-missing and matching	Non-missing and matching	More than one-month delay
	Non-missing and matching		Primary/alias match	
	One or more missing			Less than one-month delay

## Appendix 6

### LINKAGE ALGORITHM DESCRIPTION

The matching algorithm iteratively searches through dispensing records, finding the closest matching prescription record and subsequently removing it from future iterations, for each person and medication class keyword. The medication class keyword is generated by identifying the key active ingredients in a medication that are common between both generic and brand name equivalents, using a domain-knowledge look-up table.

Starting with the first dispensing record, all candidate prescription record links (linkage weight over the threshold and prescription date up to a maximum of six months prior to dispensing) are identified. The most recently prescribed candidate link for the dispensing is selected as the most likely match, using highest linkage weights to break ties, and the non-selected candidate links for both the matched dispensing record and the matched prescription record are excluded from future iterations. The process repeats until every dispensing record has been considered, although it is possible that no candidate links will be available for some dispensing records at later iterations if all initial prescription candidates have been successfully matched to other dispensing records.

## Abbreviations

BTS: British Thoracic Society
COPD: Chronic Obstructive Pulmonary Disease
GP: General Practitioner
ICS: Inhaled Cortico-Steroids
LABA: Long-Acting Β_2_-Agonist
LAMA: Long-Acting Muscarinic Receptor Antagonist
LTRA: Leukotriene Receptor Antagonist
NHSBSA: National Health Service Business Services Authority
PHE: Public Health England
RCT: Randomised Controlled Trial
SABA: Short-Acting Β_2_-2-Agonist
SLS: Salford Lung Study
